# TikTok and adolescent vision health: Content and information quality assessment of the top short videos related to myopia

**DOI:** 10.3389/fpubh.2022.1068582

**Published:** 2023-01-04

**Authors:** Shuai Ming, Jie Han, Meng Li, Yan Liu, Kunpeng Xie, Bo Lei

**Affiliations:** ^1^Henan Eye Institute, Henan Eye Hospital, Henan Provincial People's Hospital, Zhengzhou, Henan, China; ^2^Henan Clinical Research Center for Ocular Diseases, People's Hospital of Zhengzhou University, Zhengzhou, China; ^3^School of Medicine, People's Hospital of Henan University, Henan University, Zhengzhou, Henan, China; ^4^School of Business, Zhengzhou University of Aeronautics, Zhengzhou, Henan, China; ^5^School of Public Health, Zhengzhou University, Zhengzhou, Henan, China; ^6^Department of Ophthalmology, The First Affiliated Hospital of Zhengzhou University, Zhengzhou, Henan, China

**Keywords:** myopia, social media, information quality, misinformation, public health, TikTok

## Abstract

**Background:**

Despite the increasing recognition of the public health value of social media platforms, TikTok short videos focusing on adolescent vision health have not received much attention. We aimed to evaluate the content, sources, and information quality of myopia-related videos on TikTok.

**Methods:**

The top 200 most-liked myopia-related videos on the Chinese version of TikTok were queried and screened on March 12, 2022. The descriptive characteristics, contents, and sources of the selected 168 videos were obtained, and their overall quality, reliability, understandability, and actionability were assessed using the validated scoring instruments DISCERN and PEMAT-A/V.

**Results:**

Medical professionals were the main source (45.8%, 77/168) of videos. Misinformation (10.1%, 17/168) was mainly attributable to for-profit organizations (20%, 3/15) and individual non-medical users (31.3%, 10/32). However, their videos enjoyed the highest numbers of “likes,” “comments,” and “shares” (*P* < 0.05). The mean reliability and overall quality regarding treatment choice were (2.5 ± 0.5) and (3.1 ± 0.9), respectively. Videos on TikTok showed relatively high understandability (84.7%) and moderate actionability (74.9%). Video producers tended to partly or fully provide information regarding management (81.5%, 137/168) and outcome (82.1%, 138/168), and to ignore or only slightly mention content related to definition (86.9%, 146/169) and signs (82.1%, 138/168). The five video sources showed significant differences in the prevalence of misleading information (*P* < 0.001), publication reliability (*P* < 0.001), overall quality (*P* = 0.039), content score (*P* = 0.019), and understandability (*P* = 0.024).

**Conclusion:**

Considering the moderate-to-poor reliability and variable quality across video sources, the substantial myopia-related content on TikTok should be treated with caution. Nevertheless, TikTok videos may serve as a surrogate or supplement for information dissemination if providers can ensure more comprehensive and accurate content.

## Introduction

Myopia has been commonly recognized as an important adolescent public health issue causing significant disease burden of vision loss (1, 2). The rapid increase in the prevalence of myopia in adolescents and young adults represents a major vision health challenge in East and Southeast Asia (3). The prevalence of myopia in Chinese children and adolescents has increased steadily from 25.7% to 46.1% between 2000 to 2015 (4). Moreover, a recent large-scale study reported that the city-level total prevalence of myopia increased to 73.4% among school-aged children and adolescents (5). Thus, prevention and management of adolescent myopia should be included in the healthcare agenda in severely afflicted counties.

Family management and care play vital roles in myopia prevention and management among children and adolescents (6, 7). Education on myopia and appropriate management of children have been reported to encourage the execution of myopia control interventions by eye care practitioners and parents (8). Recognizing the important role of education in myopia management, the World Health Organization conducted myopia education programs worldwide to facilitate the dissemination of high-quality myopia education and information regarding prevention across countries (9). Nevertheless, a social media platform with high acceptance is urgently required to deliver evidence-based information to the public, especially to adolescents.

TikTok, a short video social media platform, has continued to gain popularity among adolescents and young adults and offers significant potential benefits for public health and medical education (10). TikTok was recently described as a new plausible platform to disseminate public health information in the coronavirus disease 2019 pandemic (11, 12). Despite its promising potential, other studies began to call for attention toward a series of research agenda, such as video content quality and dis/misinformation spread (11). Studies on TikTok video quality related to different diseases have shown inconsistent results (13–15). However, the current status of misinformation prevalence related to the topics of coronavirus disease 2019 (16), prostate cancer (17), and mental health disorders (18) does not indicate an optimistic outlook.

To our knowledge, the quality of online eye-related disease videos has not yet been sufficiently investigated, especially myopia, which is a high-profile topic on TikTok. Thus, to fill this gap in the literature, this study aimed to evaluate the content and quality of myopia-related medical information provided on the TikTok platform.

## Methods

### Search strategy and data extraction

**Figure 1:**
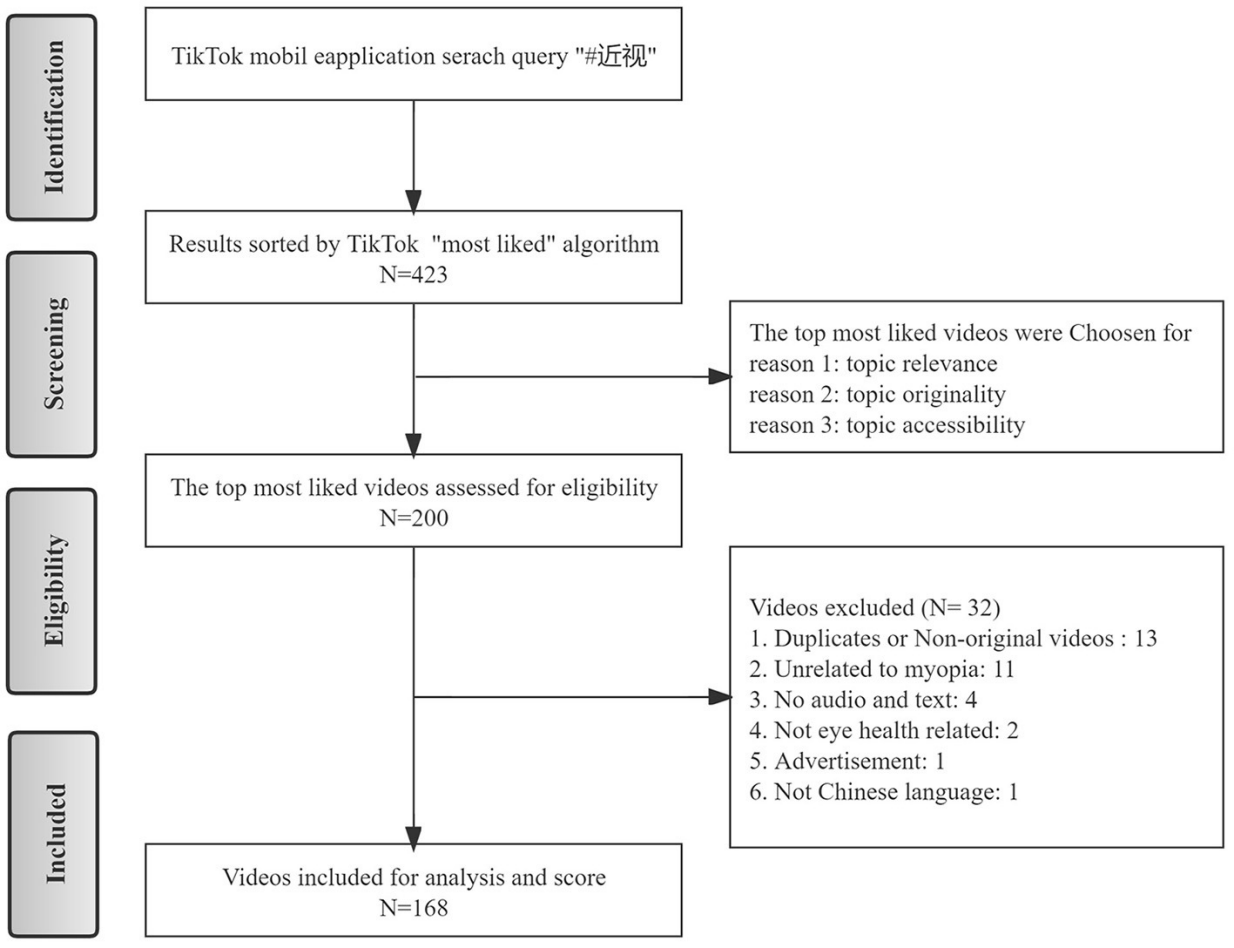
The flow chart of the inclusion method of the retrived tiktok videos.

On March 12, 2022, the keyword “近视,” which means “myopia,” was used to run a search in the search box located on the top of the opening interface of the TikTok App (Chinese version of 20.2.0). Three sort buttons, namely, “overall rank,” “most recent,” and “most likes” were provided in the search function. We assumed that users would tend to view videos with more likes; therefore, the “most likes” ranking algorithm was chosen to sort the retrieved 423 videos. Among these, videos ranked beyond 200 were less likely to be originated by the publisher and less likely to be viewed by users. Thus, only the top 200 most-liked videos were included for further eligibility evaluation. After removing unrelated, duplicated videos and those with no audio or text, we finally obtained 168 videos for data extraction and analysis (Figure 1).

Primary baseline characteristics were extracted for each video and saved in Excel format. Information included the rank on the initial TikTok search, uniform resource locator, introduction of the uploader, posted dates, length and description of each video, and the number of saves/likes/shares/comments received.

### Video source coding

Based on the authors' verification and the information provided in the video introduction, the videos were categorized into those generated by individual users and organizational users. The individual users were further categorized as medical and non-medical users. Medical users included health professionals who were verified as hospital physicians by TikTok or described themselves as health workers. Non-medical individual users were mainly general users or individual science communicators who disseminated their opinions on myopia. Organizational users included non-profit users (e.g., academic institutions, governmental accounts), for-profit users (e.g., optical shops or private sector organizations), and news agencies (19).

### Evaluation methodology

The DISCERN tool (20) and the Patient Education Materials Assessment Tool for Audiovisual Materials (PEMAT-A/V) (21) were applied for assessing video quality. The DISCERN contains 16 question items covering three aspects—reliability (8 items), the information quality about treatment choice (7 items), and the overall rating (1 items)—of the video, which are rated according to the question items. Each question is scored from 1 to 5 (question being not at all, or partially or completely answered in the video). PEMAT-A/V is a validated instrument to assess the understandability (13 items) and actionability (4 items) of audiovisual patient education. Questions are scored on a 0–1 scale that reflects disagree and agree, respectively. The final score (%) is equal to “Total points/Total possible points × 100%.”

According to the instructions, self-care is considered a form of treatment throughout the DISCERN “treatment choice” section. Therefore, we judged myopia prevention measures (more outdoor activities or less short-distance eye use, etc.) as treatment choices to avoid underestimation of the score.

We also assessed video content quality focusing on misleading information and information coverage about myopia. Content was evaluated using a predefined scoring system based on works published by the International Myopia Institute in 2019 or 2021 (22–25), which were also the standards for characterizing misleading information. Myopia video content was classified into six aspects, namely, definition, symptoms, risk factors, evaluation, management, and outcomes of myopia (see Supplementary Table 1) (19). The updated International Myopia Institute 2021 yearly digest was the main reference that represented the newest views of peer reviews (22). Diagnostic and risk content was compared with the 2019 IMI definition report and the 2021 risk factors guideline (23, 24), and management was compared with the 2019 IMI white paper on myopia clinical management guideline (25). Each aspect was scored based on a three-item scale, with a score of 0 indicating “not even addressed,” 1 indicating “partially addressed,” and 2 indicating “sufficiently addressed.” Considering some aspects involved several criteria, we defined “sufficient” when ≥3 criteria were addressed in the video.

The evaluations were performed by one ophthalmologist and one eye public health physician independently (XK and MS), based on the official handbooks. Two raters came to a consensus in understanding these constructions from beginning. The raters were only allowed to see the videos during the evaluation process. Video authors' information and classification were concealed to avoid selection bias. The intraclass correlation coefficient of the total DISCERN score between the two raters was 0.957 (0.931–0.972, *P* < 0.001). Discrepancies, if any, were resolved by discussion.

### Statistical analysis

Statistical analysis was performed using IBM SPSS Statistics 19.0 software. Data were described as frequency (percentage, %), mean (standard deviation, SD), or median (inter-quartile range, IQR), as appropriate. The kappa statistic and intraclass correlation coefficient were calculated to appraise inter-rater reliability for the source video classification and the total DISCERN score. Score differences among video source categories were tested using one-way analysis of variance coupled with the LSD-t test for the *post-hoc* test, or the Kruskal–Wallis H test followed by Bonferroni's correction on the basis of the variables' characteristics. Misleading prevalence differences related to two or more groups were tested with Chi-square test or Fisher's exact test. Pearson or Spearman rank correlation were used to explore the association of variables of content with DISCERN scores. A *P* < 0.05 was considered significant.

## Results

### Basic video characteristics

In this study, the included 168 myopia videos gathered 2,246,062 likes, 101,582 comments, and 352,365 shares after being posted for a median of 230 (164–307) days on TikTok. The videos were mainly posted by individual users (109/168, 64.9%), especially by medical professionals (77/168, 45.8%). The agreement between the raters on the source classification was 93.5%, with a kappa of 0.906 (*P* < 0.001). The median video length was 60 s, of which videos uploaded by medical professions was shortest, only 49 s. Video posted by for-profit organizations and non-medical users had higher user interactions of likes, comments, and shares (*P* < 0.05). The detail characteristics by the different sources were described in Table 1.

**Table 1:** Basic characteristics by the source of videos.

**Source of videos**	**Subgroup**	***N* (%)**	Length (Sec)^*^	Days posted^*^	*N* of likes^*^	*N* of comments^*^	***N* of shares**	*N* of saves^*^
			**Median (IQR)**	**Median (IQR)**	**Median (IQR)**	**Median (IQR)**	**Median (IQR)**	**Median (IQR)**
Organizational user	Non-profit	32 (19.1)	50 (33–89)	228 (176–276)	1,049 (553–4,621)	18 (6–70)	243 (98–616)	52 (26–115)
	For-profit	15 (8.9)	104 (73–182)	259 (160–427)	3,659 (2,204–23,537)	223 (38–943)	647 (69–5,317)	274 (23–3,966)
	News agencies	12 (7.1)	83 (46–149)	314 (223–494)	549 (271–29,114)	25 (3–1,042)	659 (13–4,276)	98 (11–717)
Individual user	Non-medical	32 (19.1)	59 (42–143)	239 (189–441)	9,715 (1,469–30,397)	107 (60–1,533)	433 (120–4,086)	228 (31–980)
	Medical	77 (45.8)	49 (34–75)	213 (106–260)	864 (373–2,371)	65 (23–177)	172 (74–547)	67 (25–141)
Overall		168 (100)	60 (35–90)	230 (164–307)	1,554 (530–5,096)	69 (20–230)	243 (91–903)	73 (27–239)

* Characteristics were significantly different in the five source subgroups. IQR, interquartile range.

### Prevalence of misinformation

A total of 19 descriptions were determined as misinformation in 17 videos. The total crude prevalence was 10.1% (17/168), and the prevalence of misinformation differed significantly among the five subgroups (*P* < 0.001). For-profit organizations (20%, 3/15) and individual non-medical users (31.3%, 10/32) were the main sources of misinformation. They tended to deliver some absolute descriptions or use terminology that was not sufficiently rigorous in their videos (see Table 2).

**Table 2:** Misinformation categorized by video numbers.

**Video series number**	***N***	**Descriptions that were not accurate or sufficiently rigorous**
14, 17	2	Too absolute: artificial light, other than asthenopia, causes myopia
22	1	All refractive surgeries cause thinning of the cornea
25, 160, 172	3	Myopia can cause hearing loss
28, 32	2	Myopia other than asthenopia can be alleviated
32	1	Too absolute: no eyedrops (without excluding atropine) are helpful for myopia
44	1	Myopia is recoverable
55, 57, 95	3	Sweet food or sodas cause myopia as being introduced in the whole video
72	1	The axial lengths, other than the diopter, that determines the definition of high myopia
96	1	The author refused to believe the fact of myopia irreversibility
104	1	Median myopia (−3.00 to −6.00 D) can recover to emmetropia with preventive methods.
105	1	The reason for myopia is an excessively thick crystalline lens
141	2	(1) Incorrect figures. (2) Correct vision care habit is the only preventive method for myopia

### Information quality

**Figure 2:**
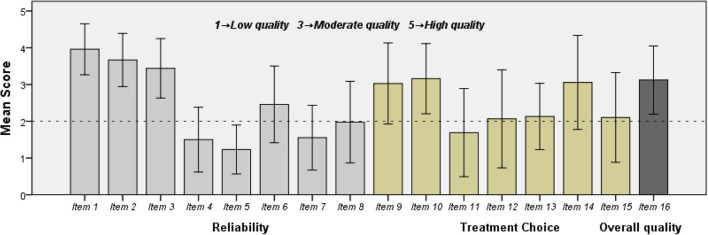
The mean DISCERN score for each item. Error bars: +/− 1 SD (standard devision). Item 1. Clear aims. Item 2. Achieves aims. Item 3. Relevant. Item 4. Clear sources of information. Item 5. Clear date of publication. Item 6. Unbiased. Item 7. Provides additional sources. Item 8. Describes areas of uncertainty. Item 9. Describes mechanism of action for treatment. Item 10. Describes benefites of treatment. Item 11. Describers risks of treatment. Item 12. Describes what would happen without treatment. Item 13. Describes how treatment would affect life quality. Item 14. Deacribes atternative treatment. Item 15. Supports shared decision making. Item 16. Overall quality regarding treatment choices.

The full descriptive characteristics of different sources and *post-hoc* analysis are shown in Tables 3, 4. Publication reliability was evaluated by DISCERN items 1–8. The total and mean reliability score were (19.8 ± 4.4) and (2.5 ± 0.5), respectively. Organizational users showed higher reliability than individual users (*P* = 0.001). Non-profit organizations and news agencies published videos with higher reliability than for-profit organizations and non-medical and medical individuals. The information quality of treatment choices was assessed by DISCERN items 9–15. A total of 107 (63.7%) videos provided treatment choice information. The total and mean treatment choice score were 17.2 ± 4.1 and 2.5 ± 0.6, respectively. Although non-profit organization users received the highest score, the five subgroups showed no significant differences (*P* = 0.073). Item 16 of the DISCERN tool provided an overall quality score to each video that involved treatment choice. Overall, the score showed a moderate quality score of 3.1 ± 0.9. The mean DISCERN score for each item is shown in Figure 2. Non-profit organization users and medical individual users published higher-quality videos than non-medical individual users (*P* = 0.010 and 0.018).

**Table 3:** Videos content and quality scores by source.

**Variable**	**Overall (*n* = 168)**	**Organization users**			**Individual users**		***P_1_* value**	***P_2_* value**
		**Non-profit**	**For-profit**	**News agencies**	**Non-medical**	**Medical**		
		**(*n* = 32)**	**(*n* = 15)**	**(*n* = 12)**	**(*n* = 32)**	**(*n* = 77)**		
Misinformation, *n* (%)	17 (10.1)	1 (3.1)	3 (20.0)	0 (0)	10 (31.3)	3 (3.9)	0.423	< 0.001
DISCERN total scores (/80), mean (SD)	38.2 (7.6)	41.4 (6.6)	36.7 (11.0)	40.3 (5.9)	35.1 (8.5)	37.8 (7.0)	0.051	0.094
Publication reliability (*n* = 168), mean (SD)	19.8 (4.4)	22.1 (3.4)	18.9 (5.9)	22.3 (3.0)	17.6 (4.6)	19.5 (3.9)	0.001	< 0.001
Treatment choice (*n* = 107), mean (SD)	17.2 (4.1)	19.0 (3.8)	15.9 (5.1)	16.6 (4.2)	15.5 (4.8)	17.4 (3.5)	0.303	0.073
Overall quality score (*n* = 107), mean (SD)	3.1 (0.9)	3.4 (0.7)	2.7 (1.1)	3.0 (1.1)	2.7 (1.0)	3.3 (0.9)	0.786	0.039
Low, *n* (%)	27 (25.2)	3 (13.6)	4 (44.4)	2 (25.0)	8 (47.1)	10 (19.6)	0.490	0.046
Moderate, *n* (%)	41 (38.3)	7 (31.8)	4 (44.4)	3 (37.5)	5 (29.4)	22 (43.1)		
High, *n* (%)	39 (36.4)	12 (54.5)	1 (11.1)	3 (37.5)	4 (23.5)	19 (37.3)		
PEMAT-A/V understandability, mean (SD)	84.7% (11.7%)	89.3% (10.9%)	81.2% (13.7%)	88.4% (12.4%)	80.8% (12.5%)	84.4% (10.6%)	0.050	0.024
PEMAT-A/V actionability, mean (SD)	74.9% (38.0%)	86.7% (26.4%)	73.3% (40.2%)	69.4% (43.7%)	63.8% (41.8%)	75.8% (38.5%)	0.220	0.189
Total content (/30), Mean (SD)	4.9 (2.0)	5.6 (2.2)	5.8 (2.3)	5.1 (1.7)	4.7 (2.2)	4.5 (1.6)	0.001	0.019
Reported 1–2 contents, *n* (%)	55 (32.7)	8 (14.5)	3 (5.5)	2 (3.6)	11 (20.0)	31 (56.4)	0.007	0.050
Reported 3–4 contents, *n* (%)	92 (54.8)	17 (18.5)	8 (8.7)	9 (9.8)	15 (16.3)	43 (46.7)		
Reported 5–6 contents, *n* (%)	21 (12.5)	7 (33.3)	4 (19.0)	1 (4.8)	6 (28.6)	3 (14.3)		

P_1_ represented the significance of the difference between organization users and individual users. P_2_ represented the overall significance of the difference in five subgroups. SD, standard deviation.

**Table 4:** The *post-hoc* analysis for exploring the underlying differences between video sources.

	**Sources**	**Non-profit**	**Profit**	**News agencies**	**Non-medical**	**Medical**
Overall quality and publication reliability	Non-profit	—	**0.040**	0.274	**0.010**	0.504
	For-Profit	**0.012**	—	0.448	0.958	**0.074**
	News agencies	0.881	**0.031**	—	0.363	0.459
	Non-medical	**< 0.001**	0.312	**0.001**	—	**0.018**
	Medical	**0.003**	0.574	**0.029**	**0.025**	—
Understandability and total content score	Non-profit	—	**0.025**	0.825	**0.003**	**0.044**
	For-profit	0.730	—	0.105	0.912	0.323
	News agencies	0.429	0.332	—	0.051	0.259
	Non-medical	0.068	0.071	0.572	—	0.137
	Medical	**0.005**	**0.013**	0.289	0.510	—

Overall quality and understandability were light gray highlighted; Publication Reliability and Total content score were dark gray highlighted. Bold values means the significant differences were found between each pairs of subgroups (*P* < 0.05).

This sample showed relatively high understandability (84.7%) and moderate actionability (74.9%) as assessed by the PEMAT-A/V tool. Moreover, the understandability of videos produced by non-profit organizations (89.3%) was higher than that produced by for-profit organizations (89.3% vs. 81.2%, *P* = 0.025) and non-medical individuals (89.3% vs. 80.8%, *P* = 0.003).

### Video content

**Figure 3:**
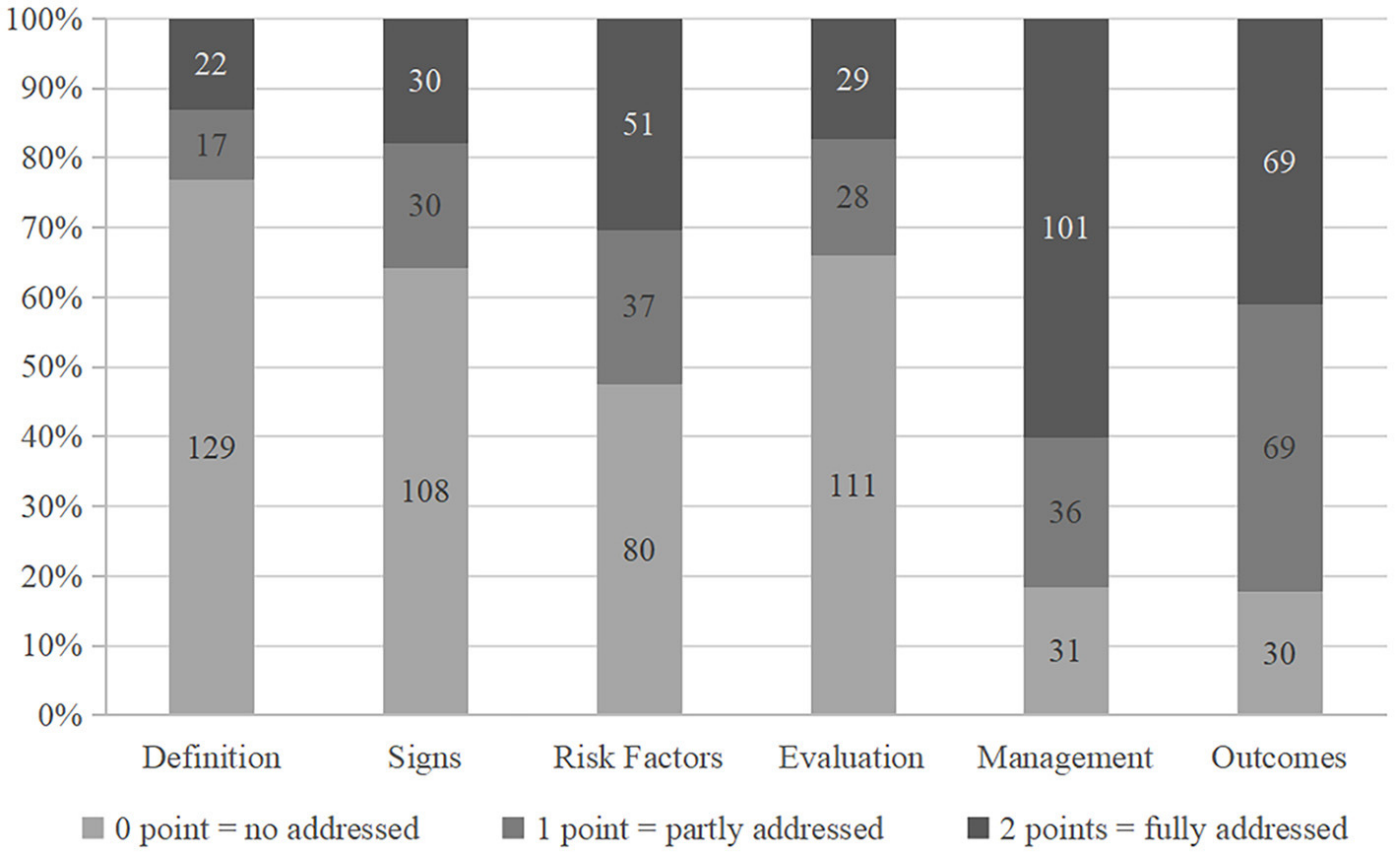
The proportion of videos addressing each content of myopia.

Figure 3 shows the degree to which each video addresses six predefined content areas (0 = no, 1 = partly, 2 = fully). More than 80% of the videos partly or fully addressed information regarding “management” (81.5%, 137/168) and “outcomes” (82.1%, 138/168) of myopia. Moreover, 60.1% (101/168) of the videos fully addressed the “management” area. However, for the “definition” area, the proportion of “not even addressed” was high (76.8%, 129/168) (see Figure 3). Overall, more than half (54.8%, 92/168) of the videos addressed 3–4 contents, and only 12.5% (21/168) of the videos addressed 5–6 contents (see Table 3).

**Figure 4:**
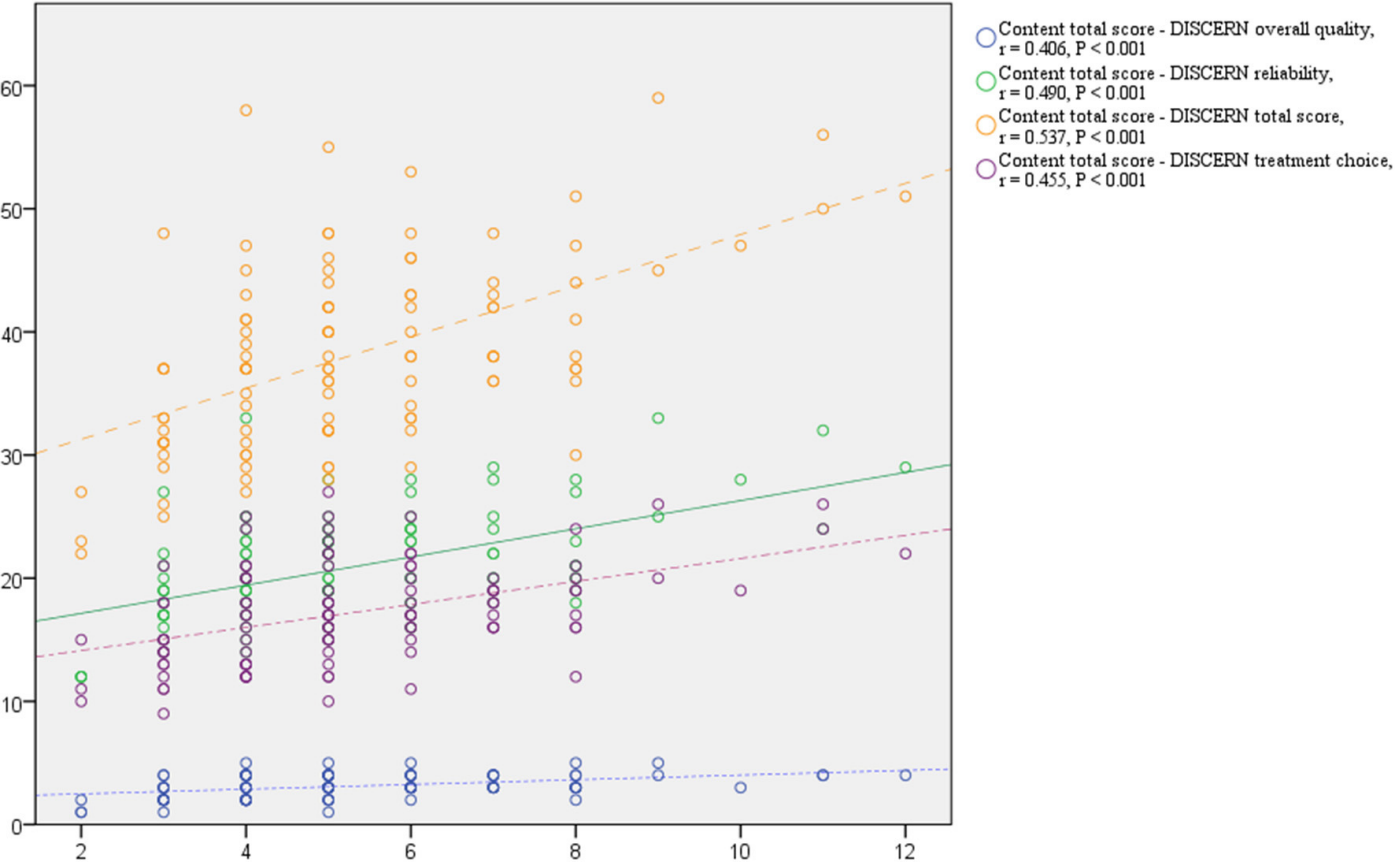
Scatter plot of DISCERN scores and content score. r, coefficient of pearson product-moment correlation.

The total myopia video content scores were different both between the two main groups (*P* = 0.001) and in the five subgroups (*P* = 0.019). Medical individual users received the lowest (4.5 ± 1.6) content score and had lower scores than non-profit (*P* = 0.005) and profit organizational users (*P* = 0.013) (see Table 4). The total content score was positively associated with DISCERN quality variables of reliability (*r* = 0.490, *P* < 0.001), treatment choice (*r* = 0.455, *P* < 0.001), overall quality (*r* = 0.406, *P* < 0.001), and the DISCERN total score (*r* = 0.537, *P* < 0.001) (see Figure 4).

## Discussion

Considering the existing barriers to promoting child and adolescent vision health in families (6), popular social media apps like TikTok can be easily accessed by parents and young people to receive myopia information. Some previous studies have evaluated adolescent health issues related to TikTok, such as social media addiction (26), vaping content (27), mental health/disorder among adolescents (28, 29), and pediatric urology (30). However, TikTok videos on adolescent vision health have been seldom evaluated. Our study is the first to describe the content and assessed information quality of short, easy-to-view myopia videos available on TikTok.

### TikTok as a source of health information

The fact that these videos amassed ~ 2.25 million likes and 102,000 comments in < 1 year highlighted the potential of TikTok as a source of vision health information for adolescent and their parents. However, in comparison with non-medical individuals and for-profit organizations, credible medical professionals who contributed to most of the myopia videos (45.8%) received the fewest likes, comments, shares, and saves. One possible explanation might be the poorly designed forms of expression in these videos (15). Most ophthalmologists uploaded videos in a lecture-like narrative form or even videos of casual dialogue during medical activities. This form of expression, which was less vivid and lacked design, was less likely to gain popularity. As the main source of myopia-related videos, health professionals should aim to create attractive educational videos, for example, by adding trending hashtags and background sounds (31), using rich supplementary visuals (lively images or realistic figures) (32), and enhancing well-organized communication skills to increase the interaction with viewers.

### Misinformation

A few previous studies have reported the prevalence of misinformation in TikTok videos, with the prevalence largely varying from 10.6% to 77.8% for various health conditions, including genitourinary cancers (36.1%, *n* = 61) prostate cancer (41.2%, *n* = 17) (17), attention-deficit/hyperactivity disorder (52%, *n* = 100) (18), mask use during the coronavirus disease-19 pandemic (10.6%, *n* = 75), (16) and pediatric urological disease (77.8%, *n* = 27) (33). The low prevalence of 10.1% identified in the present study could be attributed to the following reasons. First, most of the myopia videos (65%) were sourced from board-certified optometrists or ophthalmologists or non-profit organizations, which had the least possibility of spreading misleading information (18). Second, based on the inclusion criteria, we chose scientific educational videos that tended to contain less misinformation with high quality (34, 35). Third, information that slightly deviated from IMI standards, such as that related to eye exercises or myopia classification, was not determined to be misinformation in line with people's habits.

Although the misinformation identified in myopia-related TikTok videos was not too much and not dangerous, efforts are still needed to stop misinformation spread by for-profit organizations and individual non-medical users, since these sources published videos with higher levels of user interaction in the form of likes, comments, and shares and unfortunately contained more misinformation as well. Therefore, we strongly suggest video producers to update their knowledge according to the latest guidelines or consensus published by trusted authorities. Myopia yearly digests being published every 2 years by IMI, could be good references.

### Content of myopia-related TikTok videos

More than two-thirds of the videos referred to at least three of the six contents. The majority (>80%) of myopia videos partly or fully addressed the “management” and “outcomes” content. In comparison, “definition” and “sign” content was ignored or only slightly discussed in most videos (>80%). This imbalance has been reported in diabetes and genitourinary cancer-related disease (13, 15). The results also highlighted the challenge in providing full-content information in a 60-s TikTok video. Among the videos prepared by medical professionals, 56.4% only reported 1–2 contents. Information providers were more likely to advise viewers on preventing and controlling myopia instead of emphasizing the definition of myopia and its symptoms. Since the “sign” content (especially for high myopia) is far more complex than the general public knowledge level, videos with more comprehensive educational content are still urgently needed. Thus, medical professionals should aim to refine their video content by highlighting the complexity of myopia and the importance of early regular evaluations by an optometrist or ophthalmologist.

### Quality of myopia-related TikTok videos

Similar to the findings for most other topics, (15, 17, 32, 34–36) myopia-related TikTok videos had moderate-to-low quality in our study. The low reliability and poor treatment choice score might be explained by the poor DISCERN scores for items 4, 5, 7, and 11 (as shown in Figure 2), which implied that information providers rarely reported references to the sources used as evidence. Without clear sources and publication dates of the information, it was difficult to guarantee good reliability, even if the educational aims were clear and highly achieved. The risks or side effects were also largely ignored when treatment choices were suggested to the public. Video producers should aim to fill these gaps to ensure dissemination of evidence-based information to parents and adolescents.

Moreover, information quality varied across video sources. Non-profit organization published videos with the highest reliability and overall quality. In comparison, videos from non-medical individuals scored the lowest. Medical individuals have a natural advantage in propagating accurate educational information. However, their videos were unreliable without reporting the information sources. These results provided important insights for utilizing TikTok as a platform for vision health communication among adolescents and young people. Medical practitioners should strengthen their cooperation with non-profit organization and news agencies to better deliver educational information with high quality. Nevertheless, adolescents and parents should exercise caution while viewing videos uploaded by non-medical individuals or for-profit organizations.

The PEMAT-A/V score of 85% for understandability was relatively high. This suggested that most of the video content was presented in an understandable manner. A score of 75% for actionability was also higher than those for mental disorder (18) and prostate cancer videos (17). This might be explained by the high proportion (>80%) of “management” content addressed in myopia-related videos. These videos were likely to provide suggestions to parents and adolescents about actions for myopia prevention.

### Limitations

First, we chose the top “liked” videos with high popularity as the analysis sample, since videos with higher “likes” implicitly attract more attention. However, this was based on the assumption that viewers sorted the TikTok algorithm-recommended videos by the “most liked” label before watching the content, which might be not completely representative. The public still might directly browse the default TikTok searching list without performing other operations. Therefore, we will apply this sample-selection strategy in future studies. Second, based on the nature of cross-sectional studies, the current results only represented the condition at the time point when we captured the sample, but these results will vary over time on a dynamic platform like TikTok. Thirdly, although the DISCERN tool has been previously applied for analysis of the quality of Youtube (19), Twitter, and TikTok videos, this tool was mainly developed for written material. In our final analysis, the strong positive associations with content measures ascertained its reasonable use for TikTok videos. Finally, all videos in the Chinese version of TikTok were presented in Mandarin, precluding analysis of videos in English and other languages, which could undermine the external validity of this study. Despite these limitations, as the first quality assessment of TikTok myopia-related videos, our study still provides first-hand information on this topic.

## Conclusion

Myopia-related TikTok videos with high interactivity and a low prevalence of misinformation could be a surrogate or supplement for medical information outreach. However, the most popular videos on the platform were not sourced from mainstream medical professionals. Considering the moderate-to-low reliability and variable quality across video sources, parents and adolescents should exercise caution while reviewing the large body of myopia-related information on TikTok. Video providers, especially medical professionals, are responsible for creating more comprehensive and accurate content to satisfy public information requirements.

## Data availability statement

The original contributions presented in the study are included in the article/Supplementary material, further inquiries can be directed to the corresponding authors.

## Author contributions

SM designed the study and drafted the manuscript. SM, JH, and KX collected all relevant data and assisted in results interpretation. SM and ML carried out data analysis. BL conceived the study. YL, KX, and BL participated in the design and coordination. All authors contributed to the article and approved the submitted version.
