# Supplementary material for: TikTok and adolescent vision health: Content and information quality assessment of the top short videos related to myopia
Source: Front Public Health. 2023 Jan 4;10:1068582. doi: 10.3389/fpubh.2022.1068582 (PMC9845771; doi:10.3389/fpubh.2022.1068582)
Supplement: Supplementary file 1 [file Table_1.DOCX]

**Supplemental Table E1. The content categories and criteria of myopia-related video**

| **Category** | **Criteria** | **Examples** |
| --- | --- | --- |
| **Definition** | Qualitative or  Quantitative definition | refractive error, Rays of light entering the eye parallel to the optic axis are brought to a focus in front of the retina, ≤ -0.50 D, greater axial length, overly curved corneal, increased optical power, etc. |
| **Signs/Symptoms** | Vision symptoms,  Ocular-comfort signs | Blurred vision, Hazy vision, Glare or light sensitivity, ghost images around objects, Double vision, Haloes, Starbursts, Colour distinction problem; Dryness, Tearing, Eye discharge, etc. |
| **Risk Factors** | Major Factors, Basic birth factors, other personal factors, Family Characteristics, Environment, Common Beliefs, Genetics, others | Education, Nearwork, Time Outdoors, Screen time, Computer and Smart Phone use; Age, Sex, Ethnicity, Birth order, Birth Season; Height, Intelligence, Physical Activity, Sleep; Socio-economic Status, Smoking, Diet; Urban/Rural, Housing, Pollution, Circadian Rhythms, Night light, light spectrum; Fertility treatment, Febrile diseases, Reading in dim light, under bed-clothes or in transport, etc. |
| **Evaluation** | Standard examination, Visual habits and environment evaluation, Binocular vision evaluation, Dry eye evaluation, Accom- modative/Vergence function test, others | History taking, Parental history, history of progression, previous control treatment, Refraction, BCVA, Slit-lamp, Corneal topography, AL, IOP; IOL Master, LENSTAR, Fundus examination and imaging; Daily average hours of time spent near work and outdoor; accommodative accuracy, accommodative amplitude or the maximum accommodative ability, Distance and near heterophorias; Relative Peripheral Refraction, Higher-Order Aberrations, SCT, etc. |
| **Management** | Prevention, Progression Control and intervention, drug/surgery treatment | Myopia screening and surveillance, Education, Less Near work Activity, More Outdour activity, Myopia correction, Topical Atropine, SCL, Multifocal Spectacle Lenses, Dual-Focus and MultiFocus Contact Lenses, LASIK, PRK, RK, OK, etc. |
| **Outcomes** | Quality of life, Vision loss, Cost or Burden | Activity limitation, emotional impact, social impact and inconvenience, Worsen vision or progression, [incurable](javascript:;) but controllable |

Abbreviations: IOP, Intraocular Pressure; AL, Axial Length; SCT, Subfoveal Choroidal Thickness; BCVA, Best-corrected visual acuity; SCL, soft contact lens; LASIK, Laser-assisted In Situ Keratomileusis; PRK, Photorefractive Keratectomy; RK, Radial Keratotomy; OK, Orthokeratology.
